# Altered Antioxidant-Oxidant Status in the Aqueous Humor and Peripheral Blood of Patients with Retinitis Pigmentosa

**DOI:** 10.1371/journal.pone.0074223

**Published:** 2013-09-12

**Authors:** Cristina Martínez-Fernández de la Cámara, David Salom, Ma Dolores Sequedo, David Hervás, Cristina Marín-Lambíes, Elena Aller, Teresa Jaijo, Manuel Díaz-LLopis, José María Millán, Regina Rodrigo

**Affiliations:** 1 Sensorineural Disorders, Health Research Institute-La Fe, Valencia, Spain; 2 Department of Ophthalmology, La Fe University Hospital, Valencia, Spain; 3 Biostatistics Unit, Health Research Institute-La Fe, Valencia, Spain; 4 Centre for Biomedical Network Research on Rare Diseases (CIBERER), Valencia, Spain; 5 Genetics Unit, La Fe University Hospital, Valencia, Spain; University of Florida, United States of America

## Abstract

Retinitis Pigmentosa is a common form of hereditary retinal degeneration constituting the largest Mendelian genetic cause of blindness in the developed world. It has been widely suggested that oxidative stress possibly contributes to its pathogenesis. We measured the levels of total antioxidant capacity, free nitrotyrosine, thiobarbituric acid reactive substances (TBARS) formation, extracellular superoxide dismutase (SOD3) activity, protein, metabolites of the nitric oxide/cyclic GMP pathway, heme oxygenase-I and inducible nitric oxide synthase expression in aqueous humor or/and peripheral blood from fifty-six patients with retinitis pigmentosa and sixty subjects without systemic or ocular oxidative stress-related disease. Multivariate analysis of covariance revealed that retinitis pigmentosa alters ocular antioxidant defence machinery and the redox status in blood. Patients with retinitis pigmentosa present low total antioxidant capacity including reduced SOD3 activity and protein concentration in aqueous humor. Patients also show reduced SOD3 activity, increased TBARS formation and upregulation of the nitric oxide/cyclic GMP pathway in peripheral blood. Together these findings confirmed the hypothesis that patients with retinitis pigmentosa present reduced ocular antioxidant status. Moreover, these patients show changes in some oxidative-nitrosative markers in the peripheral blood. Further studies are needed to clarify the relationship between these peripheral markers and retinitis pigmentosa.

## Introduction

Retinal degenerations are the major cause of incurable blindness characterized by loss of retinal photoreceptor cells. Retinitis Pigmentosa (RP) is a common form of retinal degeneration, constituting the largest Mendelian genetic cause of blindness in the developed world. It has a prevalence of 1 in 4000, and it has been estimated that about two million people are affected worldwide. Patients with RP typically loose night vision in adolescence, peripheral vision in young adulthood, and central vision later in life due to the progressive loss of rod and cone photoreceptor cells by apoptosis [1]. About 20% of patients have an associated hearing loss and the combination is called Usher syndrome. There are approximately 60 genes implicated in the pathology of RP [2], [3].

Photoreceptor cell death starts with rod photoreceptor degeneration and eventually cone cell death, which is the major problem affecting RP patients because it leads to loss of central vision. Cone cells die possibly as a consequence of progressive oxidative damage [4]–[8], metabolic dysregulation, loss of trophic support [9] and, toxicity due to rod cell death [10]. Photoreceptor cells are especially susceptible to oxidative stress because of their high metabolic rate and their environmental risks such as exposition to ultraviolet radiation or high oxygen tension. The endogenous antioxidant machinery, which includes the mitochondrial antioxidant enzymes superoxide dismutases (SOD), glutathione peroxidases and catalases [11], contributes to reduce oxidative stress in photoreceptor cells. Rods represent 95% of all photoreceptors in humans [12] and are the main consumers of oxygen in the outer retina. Rods die in early stages of the disease, leading to an overload of oxygen in the retina. The cone redox balance is then disturbed and the resulting oxidative stress exceeds the antioxidant capacity of cones, contributing to their death. The *oxidative stress hypothesis* is supported by several lines of evidence in experimental models of RP [6], [7], [13], [14]. In these models, oxidative markers such as decreased reduced form of gluthatione, increased malondialdehyde, or nitric oxide [7], [13], [15] have been found. Supporting this idea, antioxidant formulations have reduced cone cell death in models of RP [6], [7], [16], [17]. In addition, overexpression of the endogenous antioxidant enzymes, including SOD and glutathione peroxidase, decreased oxidative damage and prolonged cone survival in some RP mouse models [18], [19].

In this study, we evaluated the presence of some markers of the antioxidant-oxidant status in aqueous humor and peripheral blood of RP patients and compared them with those found in healthy controls. We measured total antioxidant capacity, extracellular superoxide dismutase (SOD3) activity, nitric oxide formation and protein concentration in aqueous humor. We also determined total antioxidant capacity, SOD3 activity, SOD3 content, cyclic GMP, nitrotyrosine, nitric oxide and TBARS formation in peripheral blood. In addition, we evaluated the relationship between visual function and ocular antioxidant status. To our knowledge, this is the first time that ocular antioxidant status has been evaluated in RP patients.

## Materials and Methods

### Participants in the Study

Fifty-six patients with typical forms of RP characterized by an elevated final dark-adaptation threshold, retinal arteriolar narrowing, and a reduced and delayed electroretinogram were enrolled in the study. Thirty-seven patients donated aqueous humor and blood and only nineteen patients donated blood. Smoking and antioxidant consumption were taken into account in the study. Sixty age-matched subjects with no confounding ocular or systemic disease (blood donors) and thirteen patients suffering from cataracts without any other ocular or systemic disease (aqueous humor donors) served as controls. Since this was a retrospective non-randomized, observational study it was difficult to know whether there were a hidden non-measured variable (diet, environmental, etc.) affecting our results. However, it is possible to look for these hidden data mentioned above, using the Rosenbaum Sensitivity test [20], that addresses the possibility of hidden bias in observational studies. This analysis may answer the question “how high an unmeasured covariate’s effect would have to be to alter the conclusions of the study”. The result of the Rosenbaum Sensitivity test showed that the differences found among groups are highly insensitive against hidden bias, and therefore that there are not hidden variable affecting our results.

The characteristics of the patients enrolled in the study, all of them Caucasian, are shown in Table 1. Written informed consent was obtained before extraction of aqueous humor and peripheral blood. The procedure complied with the Declaration of Helsinki and was reviewed and approved by the Ethics Committee of La Fe University Hospital (Valencia, Spain).

**Table 1:** Characteristics of participants included in the study.

	Aqueous humor samples		Blood samples	
	Control	RP	Control	RP
**N° of subjects**	13	37	60	56
**Males**	7	26	31	26
**Females**	6	11	29	30
**Age (yr)**	60±3	46±2	41±2	44±2
**Type of RP**	–	3 AR	–	5 AR
		7 AD		8 AD
		6 USH		15 USH
		21 unknown		28 unknown

**Note**: RP: non syndromic RP; USH: Usher syndrome; AR, autosomic recessive; AD, autosomic dominant.

Patients diagnosed with RP were recruited from *Retina Comunidad Valenciana- Asociación Afectados por Retinosis Pigmentaria* and also from the Ophthalmology Service of La Fe University Hospital (Valencia, Spain). Healthy controls were recruited from the Ophthalmology Service of La Fe University Hospital (Valencia, Spain) and also from the Biobank La Fe (Valencia, Spain).

### Ophthalmic Examination

Patients from which aqueous humor was obtained underwent a full ophthalmic examination including best-corrected visual acuity (BCVA) and automated visual field (VF). The BCVA was measured according to the Early Treatment Diabetic Retinopathy Study (ETDRS) protocol adapted for use in the Age-Related Eye Disease Study [21]. We used the measurement of static perimetric sensitivities (i.e., total point score) (30-2 program with size V target; Humphrey Field Analyzer (HFA); Carl Zeiss Ophthalmic Systems, Inc., Dublin, CA). The size V target was used to minimize the number of locations with floor effects (sensitivity, ≤0 dB). The FASTPAC test strategy was used to test the central 30-2 visual field [22], [23].

### Aqueous Humor Extraction

Aqueous humor samples from 37 RP patients (31 with non-syndromic RP and six with Usher syndrome) were collected under sterile conditions in a cabin for controlled air quality (ARCSterile) (ARCMedical, Barcelona, Spain). We applied one drop of povidone iodine applied before and after the anterior chamber was punctured using a 30-gauge needle. Antibiotic prophylaxis was subsequently administered for several days. At the time of the sample collection, six patients were smokers and 14 took some kind of antioxidant supplements including vitamins, omega-3 fatty acids, and minerals. Aqueous humor samples from 13 patients suffering from cataracts without any other ocular or systemic disease were collected with a 30-gauge needle just before the beginning of the cataract surgery and served as controls. The aqueous samples of controls were collected under the same conditions before cataract surgery began. Undiluted aqueous samples of at least 100 µL were collected from each patient, placed in sterile tubes, and stored immediately at −80°C until use. All specimens were assayed to evaluate antioxidant status in a double-blind arrangement with respect to their group. For each patient, aqueous humors were collected from the eye with the more severe retinopathy or alternatively from the eye with the worse visual acuity.

### Serum Preparation

Whole blood (8 mL) without EDTA from 56 RP patients (41 with non-syndromic RP and 15 with Usher syndrome) and 60 healthy controls was obtained and centrifuged at 2000 g for five min at 20°C. At the time of the sample collection, 10 patients were smoker and 24 took some type of antioxidant supplements. Serum was collected, aliquoted and frozen at −80°C prior to biochemical determinations were performed. To avoid cGMP degradation 7 mM EDTA was added to an aliquot before freezing.

### Evaluation of Antioxidant-oxidant Status

Antioxidant-oxidant status was analysed in aqueous humor and serum from RP patients and healthy controls. Serum samples were assayed for total antioxidant capacity (TAC), TBARS formation as indicator of malonyldialdehyde (MDA) formation, and SOD3 activity.

TAC was measured using a commercial kit (Cayman Chemical, Ann Arbor, MI) according to manufacturer’s instructions. Serum and aqueous humor TAC levels were expressed as µmol/mL.

MDA levels were detected by a colorimetric method involving thiobarbituric acid (TBA) adduct formation (Cayman Chemical, Ann Arbor, MI) according to manufacturer’s instructions. Serum TBARS levels were expressed as µmol/L.

SOD3 activity was measured using a commercial kit (Cayman Chemical, Ann Arbor, MI) according to manufacturer’s instructions. Serum and aqueous humor SOD3 activity were expressed as U/mL.

### Western Blot

Fifty µg total serum protein from 28 RP patients and 27 healthy controls were subjected to electrophoresis in a 10% polyacrylamide gel containing 0.1% SDS under reducing conditions, transferred to a polyvinylidene difluoride (PVDF) membrane, and probed with rabbit anti-human SOD3 (1∶3000, abcam plc, Cambridge, UK) or rabbit anti-human transferrin (1∶1000, abcam plc. Cambridge, UK). The images were captured using an EPSON SCAN from EPSON Corporation (EPSON IBERICA, Barcelona, Spain) and quantified using the Alpha Imager 2200 (version 3.1.2) software (AlphaInnotech Corporation, San Francisco, CA, USA). SOD3 content was normalized to transferrin, a loading control for serum samples [24].

### Cyclic GMP Determination

Serum preserved in 7 mM EDTA was used to determine the amount of cGMP. cGMP was measured by using the BIOTRAK cGMP enzyme immunoassay kit (GE Healthcare Europe GmbH, Barcelona, Spain). cGMP levels were expressed as pmol/mL.

### Nitrites and Nitrates (NOX) Determination

Nitrites (stable end-product of NO) and nitrates (NOX) levels were measured in serum by the spectrophotometric Griess reaction [25] using nitrate reductase. Serum NOX levels were expressed as nmol/mL.

### Nitrotyrosine Determination

Serum-free nitrotyrosine was measured by the analytical method LC-MS/MS system, which consisted of an Acquity Ultraperformance LC® liquid chromatography system coupled to an Acquity® TQ-S triple quadruple mass Spectrometer (Waters, Manchester, UK).

Nitrotyrosine and phenyl-alanine-D5 (internal standard) stock solutions were separately prepared at 200 µM in deionized water. Two internal standard solutions at 2 µM and 0.1 µM, and a nitrotyrosine solution at 10 µM were prepared separately in deionized water from the respective stock solutions. Subsequent work solutions were prepared by dilution 1∶1 (v/v) with deionized water of the nitrotyrosine solution (10 µM) within the concentration range from 625 nM to 0.153 nM. After thawing at room temperature, 50 µL of serum was precipitated (1∶2, v/v) with ice-cold acetonitrile (ACN), vortexed, and centrifuged at 13000 g for 15 min at 4°C. Then, 100 µL of the supernatant was evaporated to dryness using a Savant Speed Vac SPD121P concentrator (Thermo Fisher Scientific SLU, Madrid, Spain) and redissolved in 100 µL of deionized water containing the internal standard (0.1 µM). Finally, the solution was injected into the LC-MS/MS system. Mobile phase consisted of deionized water and ACN, both containing 0.1% (v/v) of formic acid (FA). The injection volume was 5 µL.

The triple quadrupole MS detector operated in positive electrospray ionization (ESI) mode by selected reaction monitoring (SRM). Calibration curves were obtained by plotting the target analyte/internal standard peak area ratio for each analyte against its added concentration. Free nitrotyrosine levels were expressed as nmol/L.

### Protein Concentration

Protein concentration was measured in aqueous humor and serum by the bicinchoninic acid (BCA) protein assay (BCA Kit; Pierce Scientific, CA). Protein content was expressed as mg/mL.

### Human Mononuclear Cell Isolation

Whole blood (4 mL) with EDTA from 20 RP patients and 20 healthy controls was obtained and diluted with one volume of phosphate-buffered saline (PBS). Peripheral blood mononuclear cells (PBMC) were obtained as described by Kimura et al. [26]. In brief, diluted blood was layered onto Lymphoprep solution (density 1.077 g/mL, AXIS-SHIELD, Oslo, Norway) and centrifuged at 800 g for 20 min at 20°C. The pellet containing mononuclear cells was collected and washed with three volumes of phosphate-buffered saline and centrifuged at 450 g for 10 min. The cellular pellet was frozen at −80°C until RNA extraction.

### RNA Extraction and cDNA Synthesis

Total RNA was isolated from PBMC using RNeasy mini kit (Qiagen, Hilden, Germany) following the manufacturer’s protocol. RNA concentration and purity were determined by UV spectrophotometry at 260 and 280 nm wavelengths. Then, cDNA was synthesized from 1 µg of total RNA by reverse transcription using the GeneAmp Gold RNA PCR Reagent kit (Applied Biosystems, Carlsbad, CA, USA) following manufacturer’s instructions.

### Quantitative Real-time PCR Analysis in PBMC

The relative expression of inducible nitric oxide synthase (iNOS) (Hs01075529_m1) and heme oxygenase 1 (HO-1) (Hs01110250_m1) was evaluated by qPCR using the TaqMan® gene expression assay (Applied Biosystems, Life Technologies Corporation, Carlsbad, California, USA). Glyceraldehyde 3-phosphate dehydrogenase (GAPDH) (Hs02758991_g1) gene was used as a housekeeping gene control. A positive control of iNOS was obtained from human umbilical vein endothelial cells (HUVEC) treated with 10 µg/mL LPS for 4 hours.

### Statistical Analyses

All statistical analyses were done using R software (version 2.15.3) (Foundation for statistical computing, Vienna, Austria). Multivariate analysis of covariance (MANCOVA) [27], fuzzy clustering [28], [29], Mann-Whitney test, Rosenbaum’s Sensitivity test [20] and multiple linear regression models were used to analyze the data. A 0.05 significance threshold was used.

## Results

### Antioxidant Status in Eyes from Patients with Retinitis Pigmentosa

Fifty aqueous humor samples were collected from 37 RP patients (31 with non-syndromic RP and six with Usher syndrome) and from 13 healthy controls. Table 1 shows demographic features of all participants enrolled in this study. Few markers could be explored because of limited availability of aqueous humor including TAC, SOD3 activity, NOX formation and protein concentration. Descriptive statistics of each marker are shown in Table 2.

**Table 2:** Antioxidant-oxidant markers and protein in aqueous humor from RP patients and healthy controls.

	Control	RP
**TAC (µmol/mL)**	1.46±0.1	1.06±0.1*
**SOD3 (U/mL)**	9.8±1.5	3.7±0.4**
**Protein (mg/mL)**	3.6±0.2	3.1±0.2
**NOX (nmol/mL)**	198±25	231±22

**Note**: RP: retinitis pigmentosa; TAC: total antioxidant capacity; SOD: superoxide dismutase; NOX: nitrates and nitrites. Values are expressed as mean ± SEM. MANCOVA was carried out considering all response variables (TAC, SOD3, Protein and NOX) simultaneously (*p<0.05; **p<0.01).

Since several markers (response variables) were measured from the same individuals and since these variables were correlated, performing analysis of covariance (ANCOVA) tests for each response variable would yield non- independent results and make the results difficult to interpret. This approach would also increase the family-wise type I error rate. A sensible approach would be to assess group differences on all the response variables considered simultaneously. Following this approach, we performed a multivariate analysis of covariance (MANCOVA) with the disease, age, gender, and antioxidant consumption as predictive variables. This revealed that RP significantly altered ocular antioxidant status in aqueous humor (p = 0.001) (Table 3). We found no statistical evidence for gender, age or antioxidant consumption effects. Further analysis of each of the response variables indicated that TAC and SOD3 activity were decreased in patients with RP (p = 0.017 and p<0.001, respectively). Stable end-products of nitric oxide, NOX, were not affected by RP (p = 0.630). However, protein concentration showed a tendency to decrease in RP patients (p = 0.057). No statistical differences (p = 0.450) were found between patients with syndromic RP (Usher syndrome) and non-syndromic RP (data not shown).

**Table 3:** MANCOVA in aqueous humor and blood from patients with RP and healthy controls.

	Aqueous humor		Blood	
PredictiveVariables	Pillaístrace	P-value	Pillaístrace	P-value
**Age**	0.1261	0.2753	0.1832	0.0119
**Gender**	0.1003	0.4043	0.1107	0.1307
**Disease RP**	0.3841	0.0010	0.2579	0.0025
**Antioxidant consumption**	0.2014	0.0737	0.0752	0.3403

**Note**: Pillaís trace, multivariate test criteria used in the MANCOVA.

Due to the large number of oxidants contained in cigarette smoke, we analyzed whether smokers (16% of patients) showed an altered antioxidant status compared to non-smokers in aqueous humor. No statistical differences were found between smoker and non-smoker patients (p = 0.490).

### Antioxidant-oxidant Status in the Peripheral Blood of Patients with Retinitis Pigmentosa

In light of these results, we assessed whether antioxidant-oxidant status was also disturbed in peripheral blood. Blood samples were collected from 41 patients with non-syndromic RP, 15 patients with syndromic RP (Usher syndrome) and 60 healthy controls (Table 1). We analyzed the following markers: TAC, SOD3 activity and content, TBARS formation (as an indicator of lipid peroxidation) and metabolites of the NO/cGMP pathway.

MANCOVA revealed alterations in the antioxidant-oxidant status in serum of RP patients (p = 0.002) (Table 3). Descriptive statistics of each response variable are shown in Table 4. This analysis indicated that age seems to play a role in serum antioxidant-oxidant status, with higher levels of oxidative-nitrosative markers in older participants than in younger participants (p = 0.012). However, we did not find statistical evidence for gender or antioxidant consumption effects. Our results revealed that while serum SOD3 activity was decreased, serum TBARS formation was significantly increased in RP patients (p = 0.025 and p = 0.004 respectively) compared to healthy controls. No significant differences were found in serum TAC.

**Table 4:** Antioxidant-oxidant markers in blood from RP patients and healthy controls.

	Control	RP
**TAC (µmol/mL)**	1.15±0.04	1.17±0.05
**SOD3 (U/mL)**	11.60±0.70	8.30±0.40*
**TBARS (µmol/L)**	5.10±0.20	7.10±0.40**
**NOX (nmol/mL)**	49±2	57±3*
**cGMP (pmol/mL)**	0.41±0.03	0.68±0.06*

**Note**: RP: retinitis pigmentosa; TAC: total antioxidant capacity; SOD: superoxide dismutase; TBARS: thiobarbituric acid reactive substances; NOX: nitrates and nitrites; cGMP: cyclic guanosine monophosphate. Values are expressed as mean ± SEM. MANCOVA was carried out considering all response variables (TAC, SOD3, TBARS, cGMP and NOX) simultaneously (*p<0.05, **p<0.01).

**Figure 1:**
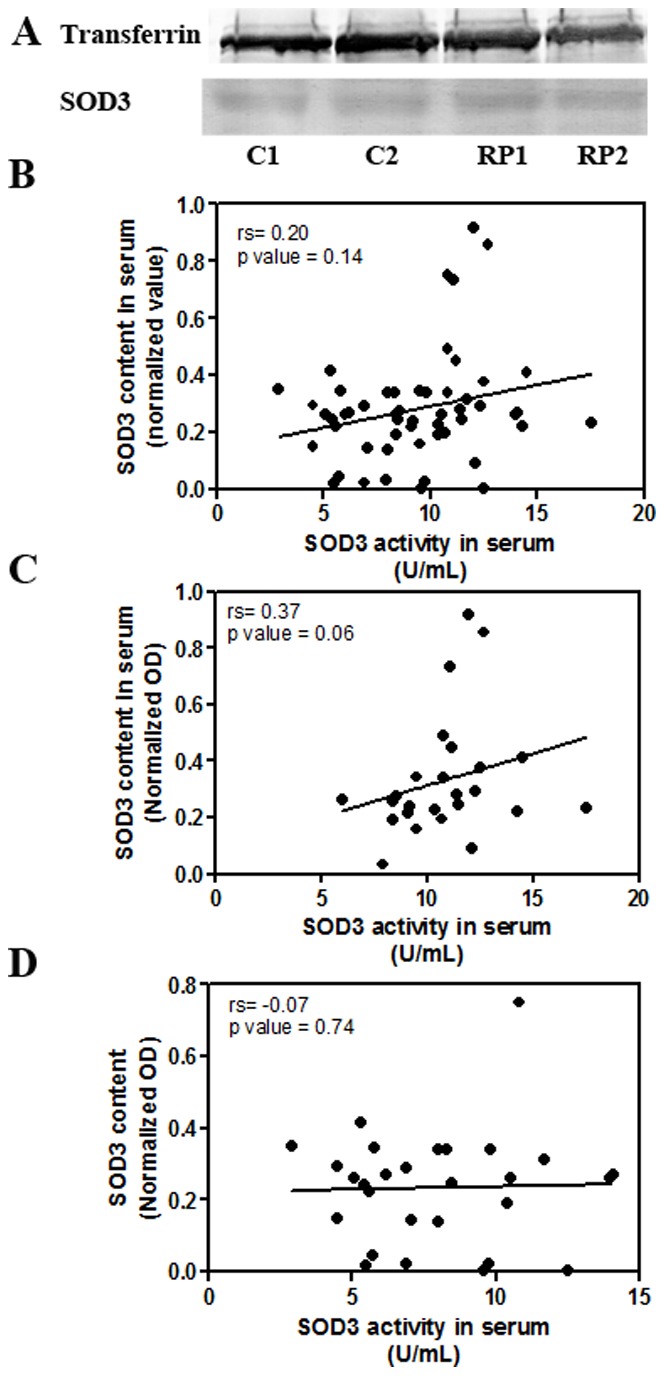
Protein content of SOD3 in serum from RP patients and healthy controls. (A) Fifty µg of total serum protein were subjected to electrophoresis and extracellular SOD3 content was analysed by inmunoblotting, as described in Materials and Methods. The immunological signal of SOD3 was normalized to transferrin, a loading control for serum samples; Correlation analysis between SOD3 activity and SOD3 content in serum from patients and healthy controls (B), healthy controls (C) or patients (D). Spearman’s correlation test was used (rs = correlation coefficient).

No statistical differences were found between healthy controls (0.33±0.04 a.u) and RP patients (0.23±0.03 a.u, Mann-Whitney test, p = 0.21) in serum SOD3 content (Fig. 1A). We analysed whether SOD3 content correlated with SOD3 activity in serum samples (Fig. 1B). SOD3 activity almost correlated with SOD3 content in healthy subjects (Spearmańs coefficient = 0.37, p = 0.06) (Fig. 1C). However, no correlation was found for RP patients (Spearmańs coefficient = −0.07, p = 0.74) (Fig. 1D).

**Figure 2:**
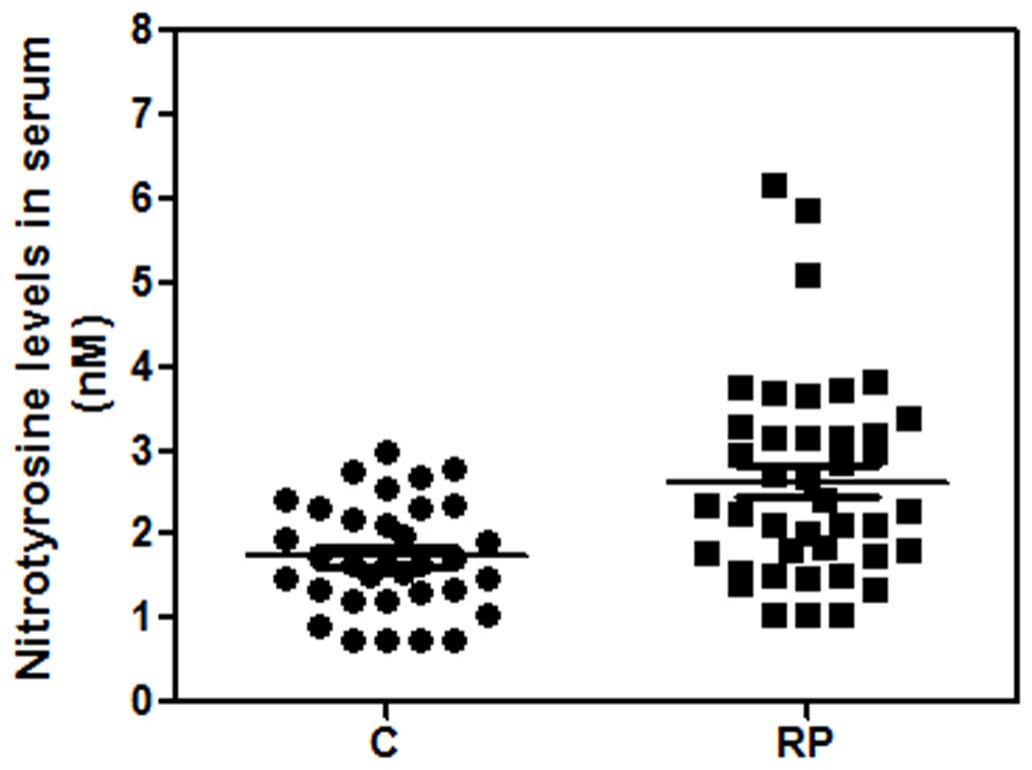
Blood free nitrotyrosine concentration in RP patients and healthy controls. Free nitrotyrosine was measured by LC-MS/MS system as described in Materials and Methods. Values are expressed as mean ± SEM.

**Figure 3:**
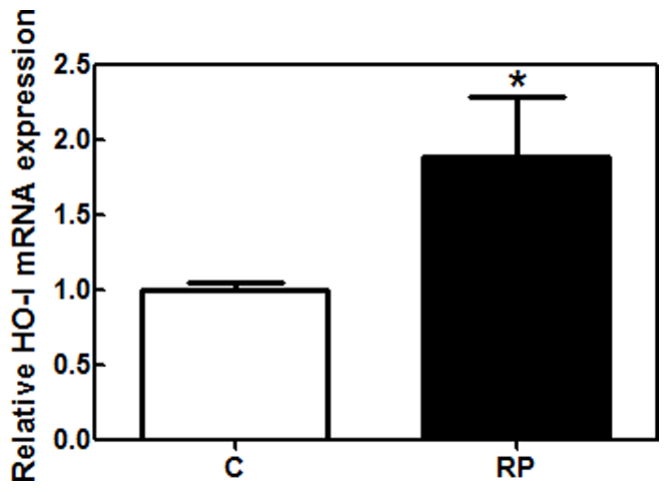
Gene expression of heme oxygenase I in peripheral blood mononuclear cells of RP patients and healthy controls. mRNA expression was quantified by qPCR analysis as described in Materials and Methods. qPCR data were normalized to housekeeping gene, *GAPDH*. C; healthy controls, RP: patients with retinitis pigmentosa; PBMC: peripheral blood mononuclear cells. Mann-Whitney test was used (*p<0.05).

The serum NO/cGMP pathway was also analyzed by measuring NOX formation, free nitrotyrosine and cGMP levels and expression of iNOS in PBMC. Serum NOX was significantly higher in RP patients (p = 0.033) than in healthy controls. Free nitrotyrosine, another indicator of NO formation *in vivo*, was not included in MANCOVA due to the high number of missing values in this variable. However, results clearly suggested that free nitrotyrosine levels were higher in RP patients (2.6±0.2 nM, n = 41) than in healthy controls (1.7±0.1 nM, n = 34) (Fig. 2). No iNOS expression was detected by qPCR in PBMC of RP patients or healthy controls. The content of cGMP, a downstream effector of NO, was higher in RP patients than in healthy controls (p = 0.015). Elevated serum cGMP could be a consequence of higher soluble guanylate cyclase (sGC) activation by NO or carbon monoxide (CO). CO is generated through heme oxygenase enzymes HO-I and HO-II. qPCR analysis revealed that HO-I was up-regulated (Mann-Whitney test, p<0.05) in PBMC from RP patients (Fig. 3).

Similar to what we found in the aqueous humor samples, there were no statistical differences between patients with syndromic RP (Usher syndrome) and non-syndromic RP (p = 0.710), and between smoker and non-smoker patients (p = 0.550) in all studied response variables (data not shown).

Taken together, these results allowed us to conclude that RP patients present reduced ocular antioxidant status that could contribute to the progression of the disease, especially to cone death. Moreover, the RP patients show, unexpectedly, imbalances in their peripheral blood antioxidant-oxidant homeostasis.

### Ocular Antioxidant Status and Visual Function in Retinitis Pigmentosa

**Figure 4:**
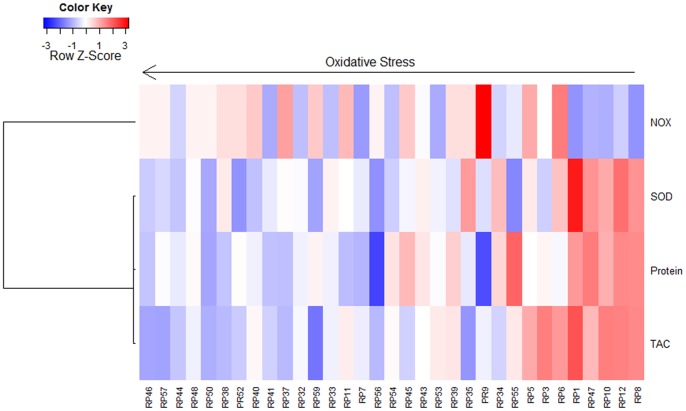
Heatmap representation of the fuzzy clustering results for antioxidant-oxidant markers in aqueous humor of RP patients. The dark red to dark blue colors correspond to high to low values. Individuals classified as with higher oxidant status show low TAC, protein and SOD3 levels and medium to high NOX levels. Individuals classified as with lower oxidant status show low NOX levels and high SOD3, protein and TAC levels.

In order to assess the possible relationship between ocular antioxidant status and best-corrected visual acuity or visual field in RP patients, we first assigned to each individual a value between 0 (lowest antioxidant status) and 1 (highest antioxidant status) computed through fuzzy clustering classification according to their NOX, SOD3, protein and TAC values (Fig. 4). The new computed variable was used as a predictor of best-corrected visual acuity and visual field in a multiple linear model including age, sex and antioxidant consumption as covariates. This revealed a strong association between the ocular antioxidant status and the visual field (p = 0.009), and suggests that RP patients with better visual field are more likely to belong to cluster 1 (highest antioxidant status). However, no effect was found on best-corrected visual acuity (p = 0.927). Analysis showed that 35% (multiple R-squared (R^2^) = 0.352) of the variability in visual field was associated to changes in the antioxidant status.

## Discussion

The retina is highly susceptible to oxidative stress because of its high oxygen consumption, its high content of polyunsaturated fatty acids, and direct exposure to light. Increasing evidence suggests that oxidative stress contributes to the pathogenesis of RP [6], [7], [18]. To our knowledge this is the first time that the level of ocular antioxidant status in RP patients has been analyzed. We determined the levels of different markers of the antioxidant-oxidant status in aqueous humor and blood from RP patients, and compared them with those in healthy controls to confirm an alteration of this status.

Recently, it has been shown that the composition of the aqueous humor is affected by the pathophysiology of RP [30] as occurs in other retinal diseases such as glaucoma [31], diabetic retinopathy [32], or age-related macular degeneration [33]. We found that the aqueous humor levels of TAC and SOD3 activity were lower in RP patients than in healthy controls. TAC and SOD are considered good biological markers of the endogenous antioxidant defence system. Therefore our results suggest that the endogenous antioxidant defence system is reduced in the eye of RP patients, and hence they have less capacity to cope with toxic oxygen intermediates.

The SODs are important antioxidant enzymes that catalyze the dismutation of the free radical superoxide into oxygen and hydrogen peroxide in many tissues, including the retina. The human eye expresses three types of SOD: the extracellular superoxide dismutase or SOD3, the cytosolic copper- and zinc-containing SOD or SOD1 and, the mitochondrial manganese-containing SOD or SOD2 [34]. SOD3 regulates extracellular concentrations of reactive oxygen species (ROS) and reactive nitrogen species (RNS) preventing the production of peroxynitrite, and possibly contributes to tissue protection during inflammatory insults [35], [36]. Therefore the reduced activity of SOD3 detected in the eye of RP patients could result in elevated superoxide [37], and SOD3 down-regulation may contribute to the inflammatory process recently described in RP [30], [38].

Concerning the other two SOD enzymes, it has been shown that retinal overexpression alone of SOD1 protected retinal cells from a severe oxidative stress induced by hyperoxia [39] but had deleterious effects in *rd1* mice [18]. However, the authors showed that combined overexpression of SOD1 or SOD2 with the peroxide-metabolizing enzyme (glutathione peroxidase 4 or catalase) within the same cellular compartment was able to reduce cone cell death in RP models [18], [19].

Numerous experimental and clinical studies indicate altered ocular blood circulation, including retinal flow [40] and choroidal circulation [40]–[43] in RP patients, possibly as a result of vascular remodelling in response to reduced metabolic demand [40], [44] and low aqueous humor VEGF-A levels [45]. Reduced aqueous humor protein concentration detected in patients could be, at least in part, a consequence of this blood flow disturbance.

Interestingly, we found that oxidative-nitrosative stress markers are high in blood from RP patients. During oxidative stress, different ROS and RNS such as superoxide, hydrogen peroxide, NO, and peroxynitrite are generated. Increased superoxide radicals resulting from decreased SOD3 activity could, in combination with high NO formation, lead to increase peroxynitrite formation in blood of RP patients. Peroxynitrite can induce lipid and protein oxidation, and nitration of proteins [46]. Nitration is the most extensive post-translational modification of proteins and can strongly affect their biological function [47]. High blood levels of nitrotyrosine could contribute to the nitration of SOD3 impairing its activity [48] without affecting the total protein content in RP patients. This could be a possible explanation for the lack of correlation between SOD3 activity and SOD3 content in RP patients. Furthermore, elevated levels of nitrotyrosine in retina of *rd1* mice correlated with cone cell death, suggesting that NO exacerbates oxidative damage to cones in RP [49].

NO is a diffusible gas synthesized from L-arginine via three different isoforms of NOS (neuronal NOS, endothelial NOS and inducible NOS). Our results demonstrated a strong tendency to up-regulate NO, free nitrotyrosine, and its downstream effector, cGMP, in the blood of RP patients. NO can act through the stimulation of the sGC with subsequent formation of cGMP. Interestingly, up-regulation of HO-I can also contribute to increasing cGMP levels in RP patients. Up-regulating the HO system should generate cytoprotective products such as bilirubin, biliverdin, and carbon monoxide (CO) that in turns should stimulate sGC [50]. On the other hand, HO induction may play an important role in the antioxidant defense system in oxidative stress-related diseases such as RP, age-related macular degeneration or in retinal damage situations such as intense light damage, retinal detachment or ischemia-reperfusion injury [51]–[53].

In blood vessels, cGMP is involved in relaxation of vascular smooth muscles leading to vasodilation and increased blood flow and inhibition of adhesion and aggregation of platelets. Systemic vascular impairment has been described in patients with RP [54]. Cellini *et al* [55] and Ohguro *et al* [56] reported that elevated levels of plasma endothelin- 1 (ET-1) which is a potent vasoconstrictive peptide found in RP patients, could lead to vasoconstriction of cutaneous capillaries. However, the increase of ET-1 in blood of RP patients is controversial [57]. The elevated cGMP can be considered as an adaptive response to vasoconstriction, or may simply be a consequence of the high NO levels associated with oxidative stress.

We found that levels of blood lipid peroxidation (measured by TBARS formation) were significantly elevated in RP patients compared with those in healthy controls, supporting previous findings [58], [59]. Retinal photoreceptor outer segment membranes are very sensitive to lipid peroxidation [60]. Lipid peroxidation injures photoreceptor cell membranes resulting in cell damage in different animal models of RP [7], [61]. It is likely, thus, that the retina of RP patients shows lipid peroxidation.

We found statistical evidence supporting an association between visual field and ocular antioxidant status in RP patients. This association seems to corroborate that an imbalance in the antioxidant machinery system is involved in the pathogenesis of RP, similar to other ocular diseases such as age-related macular degeneration, retinopathy of prematurity, retinal light damage, and glaucoma [29]–[31], [62], [63]. However, further studies are needed to analyze how antioxidant-oxidant status progresses at different stages of RP.

Under our experimental conditions, we found that patients with antioxidant consumption, including vitamins and omega-3 fatty acids, did not present higher antioxidant status than those without antioxidant consumption. Instead, they showed a trend toward diminishing TAC and SOD3 activity. However, we cannot conclude that, in this case, antioxidant consumption really worsened antioxidant status because our study was not designed to test for this. Each patient took a different kind and dose of nutritional supplement and we did not measure antioxidant status before treatment. Antioxidant supplements may have differential effects on oxidative stress responses, depending on the timing, dose and duration of the antioxidant consumption. Therefore, the effectiveness of antioxidant supplementation on antioxidant-oxidant status needs to be explored in detail. At present, the use of vitamin A and omega-3 fatty acids as appears to be an effective treatment for patient with RP, although the interpretation of clinical trials remains controversial [64]–[67]. Nonetheless several antioxidant treatments in experimental models of RP suggest a positive action of these treatments in slowing down or reducing the progression of the disease [6], [16], [68].

Concerning the effect of smoking, we did not find statistical evidence for a negative effect on the antioxidant-oxidant status in RP patients. Unfortunately there was no data for the control group but the proportion in the RP group (16% for aqueous samples and 18% for serum samples) was below average of smokers in Spanish population (35%) [69]. Therefore, if we assume that our control group represent the Spanish population this imbalance would raise mean oxidative stress in control group compared to RP group, maybe increasing type II error (false negative) but not type I error (false positive).

In conclusion, we found that RP patients present a reduced ocular antioxidant status, and an imbalance of the antioxidant-oxidant status in the peripheral blood. Therefore the eyes of the RP patients could have less capacity to cope with toxic oxygen intermediates. Although RP is a genetic disease, this reduced capacity could contribute to the progression of the degeneration, especially to cone death [7].
